# Cognitive, Emotional, and Psychosocial Functioning of Girls Treated with Pharmacological Puberty Blockage for Idiopathic Central Precocious Puberty

**DOI:** 10.3389/fpsyg.2016.01053

**Published:** 2016-07-12

**Authors:** Slawomir Wojniusz, Nina Callens, Stefan Sütterlin, Stein Andersson, Jean De Schepper, Inge Gies, Jesse Vanbesien, Kathleen De Waele, Sara Van Aken, Margarita Craen, Claus Vögele, Martine Cools, Ira R. Haraldsen

**Affiliations:** ^1^Division of Surgery and Clinical Neuroscience, Department of Medical Neurobiology, Oslo University HospitalOslo, Norway; ^2^Department of Physiotherapy, Oslo and Akershus University College of Applied SciencesOslo, Norway; ^3^Division of Pediatric Endocrinology, Department of Pediatrics, Ghent University Hospital and Ghent UniversityGhent, Belgium; ^4^Section for Psychology, Lillehammer University CollegeLillehammer, Norway; ^5^Department of Psychology, University of OsloOslo, Norway; ^6^Division of Pediatric Endocrinology, Department of Pediatrics, Brussels University HospitalBrussels, Belgium; ^7^Research Unit INSIDE, Institute for Health and Behavior, University of LuxembourgLuxembourg, Luxembourg

**Keywords:** central precocious puberty, gonadotropin releasing hormone analog, cognitive function, emotion regulation, psychosocial function, heart rate variability, puberty

## Abstract

Central precocious puberty (CPP) develops due to premature activation of the hypothalamic-pituitary-gonadal (HPG) axis, resulting in early pubertal changes and rapid bone maturation. CPP is associated with lower adult height and increased risk for development of psychological problems. Standard treatment of CPP is based on postponement of pubertal development by blockade of the HPG axis with gonadotropin releasing hormone analogs (GnRHa) leading to abolition of gonadal sex hormones synthesis. Whereas the hormonal and auxological effects of GnRHa are well-researched, there is a lack of knowledge whether GnRHa treatment influences psychological functioning of treated children, despite the fact that prevention of psychological problems is used as one of the main reasons for treatment initiation. In the present study we seek to address this issue by exploring differences in cognitive function, behavior, emotional reactivity, and psychosocial problems between GnRHa treated CPP girls and age-matched controls. Fifteen girls with idiopathic CPP; median age 10.4 years, treated with slow-release GnRHa (triptorelin acetate—Decapeptyl SR® 11.25) and 15 age-matched controls, were assessed with a comprehensive test battery consisting of paper and pencil tests, computerized tasks, behavioral paradigms, heart rate variability, and questionnaires filled in by the children's parents. Both groups showed very similar scores with regard to cognitive performance, behavioral and psychosocial problems. Compared to controls, treated girls displayed significantly higher emotional reactivity (*p* = 0.016; Cohen's *d* = 1.04) on one of the two emotional reactivity task conditions. Unexpectedly, the CPP group showed significantly lower resting heart rates than the controls (*p* = 0.004; Cohen's *d* = 1.03); lower heart rate was associated with longer treatment duration (*r* = −0.582, *p* = 0.037). The results suggest that GnRHa treated CPP girls do not differ in their cognitive or psychosocial functioning from age matched controls. However, they might process emotional stimuli differently. The unexpected finding of lower heart rate that was associated with longer duration of the treatment should be further explored by methods appropriate for assessment of cardiac health.

## Introduction

Central precocious puberty (CPP) is defined as the advent of an otherwise normal puberty before the age of 8 years in girls and 9 years in boys due to premature activation of the hypothalamic-pituitary-gonadal (HPG) axis (Nebesio and Eugster, 2007). The etiology of CPP is unclear and varies with gender. It is predominantly found in girls and while the majority of female CPP is idiopathic, in boys it is more frequently secondary to an organic cause (e.g., tumor; Choi et al., 2013). CPP incidence is age dependent. Danish data from 1993 to 2001 showed an incidence of 8:10,000 in girls aged 5–9 and 1–2:10,000 in boys aged 8–10 (Teilmann et al., 2005).

CPP is associated with early bone maturation and reduced adult height in the youngest cases. Pharmacological blockade of the gonadotropin stimulus with GnRH analogs (GnRHa), which leads to cessation of gonadal sex hormones production, is nowadays considered the standard treatment for CPP (Carel et al., 2009); the main treatment goals are an increase in adult height and prevention of psychological problems (Sonis et al., 1985; Johansson and Ritzen, 2005; Tremblay and Frigon, 2005). While research shows that treatment can positively influence adult height in treated girls, especially if started before 6 years of age (Carel et al., 2009), the effects in boys and with regard to psychological functioning are less explored. Both a recent consensus statement and an update on the usage of GnRHa in CPP strongly emphasize the need for more research regarding GnRHa effects on psychological functioning (Carel et al., 2009; Chen and Eugster, 2015).

GnRHa treatment can potentially influence CPP children's psychological functioning through several pathways. Firstly, postponement of the pubertal development by blockade of sex hormones production can reduce psychological distress associated with early biological maturation. Secondly, abolition of sex hormone influences on the developing brain may on its own have an effect on cognitive development. Finally, GnRHa can potentially influence cognitive development via GnRH receptors that are widely present in brain areas not related to reproduction (Skinner et al., 2009). Several human and animal studies suggest that GnRHa may indeed influence cognitive functioning. A decline in working and episodic verbal memory associated with GnRHa treatment has been observed in women with benign leiomyomata uteri and endometriosis (Grigorova et al., 2006; Craig et al., 2007). In an animal study, using an ovine model of pubertal development, prepubertal GnRHa treatment significantly affected emotion regulation capacity, reward seeking behavior, and emotional reactivity in young sheep (Wojniusz et al., 2011; Evans et al., 2012). Furthermore, GnRHa treatment significantly and sex-specifically affected hippocampus and amygdala gene expressions and altered amygdalae volumes in the same animals (Nuruddin et al., 2013a,b,c). In addition, possible effects of GnRHa on cardiac health have recently been postulated, following findings of increased prevalence of cardiovascular disease in prostate cancer patients treated with GnRHa (Tsai et al., 2007; Keating et al., 2010).

The consensus statements and findings from adult and animal studies warrant a broader investigation of cognitive and emotional functioning in GnRHa treated CPP children. In the current study we, therefore, compared CPP girls under GnRHa treatment to age-matched controls. We assessed children's cognitive function by using a comprehensive neuropsychological test battery consisting of paper and pencil and computerized tests. Additionally, we assessed cognitive, social, and behavioral function at home and school situations by employing questionnaires completed by the children's parents. Since animal twin studies indicated poorer emotional regulation capacity and higher emotional reactivity in GnRHa treated lambs compared to their untreated twins (Wojniusz et al., 2011; Evans et al., 2012), assessment of emotional processing was additionally included in the study. We employed the emotional flanker task (EFT) for the assessment of emotional reactivity (Bishop et al., 2004) and calculated vagally mediated heart rate variability (HRV) as a measure of emotional regulation capacity (Appelhans and Luecken, 2006; Thayer and Lane, 2009; Koval et al., 2013).

## Materials and methods

### Participants

Clinical records of girls with idiopathic CPP, treated with GnRHa between November 2009 and December 2011, either at the University Hospital Ghent or the University Hospital Brussels, were reviewed. CPP was defined according to the combination of the following three items: (a) the onset of breast development before the age of 8 years; (b) accelerated growth velocity in the months before diagnosis; and (c) advancement of bone age by at least one year compared to chronological age. In cases with uncertain diagnosis, a standardized LHRH test (applied in 12 out of 15 girls) yielding an LH peak above 4.5 U/l and the finding of an estrogenized uterus (corpus length/cervix length > 1) on pelvic ultrasound were considered as additional evidence for the presence of CPP. A minimum age of 9 years (due to the complexity of the test package), treatment by GnRHa for at least 6 months, and 2–3 monthly clinical follow-up was mandatory to enter the study.

GnRHa treatment was adjusted in case of incomplete pubertal suppression as judged by physical examination and LH/FSH blood sampling or repeated GnRH testing. At the time of the study, puberty suppression was determined clinically and radiologically as successful in all patients based on Tanner stage (no progression of breast development), growth velocity (decreased as compared to pre-treatment), and bone age. Exclusion criteria were additional endocrine or other chronic diseases, which could influence cognitive and behavioral function; learning difficulties, defined as an IQ < 70; and non-European descent due to race/ethnicity based differences concerning age of pubertal onset (Biro et al., 2013). On the basis of these criteria, two girls out of 17 were excluded. Fifteen healthy controls, carefully matched for age, were recruited through flyers distributed in public places. All patients and controls gave their assent, and parents gave written informed consent. The study was approved by the ethical committees of both institutions; *Commissie Medische Ethiek UZ Gent* and *Commissie Medische Ethiek UZ Brussel*.

### Procedures

Patients and controls were invited to either the University Hospital Ghent or Brussels. After assessment of medical history and physical examination including anthropometrics and pubertal staging by an experienced pediatric endocrinologist, bone age was assessed from an X-ray of the left hand and wrist by one single investigator (MC) according to the Greulich and Pyle method. All CPP patients and controls underwent neuropsychological assessments, an emotional reactivity test, and heart rate monitoring for calculation of HRV. Behavioral questionnaires were completed by parents. All neuropsychological tests were applied by one single psychologist (NC), experienced in pediatric clinical psychology, and trained in test administration and scoring, and consisted of a range of cognitive, behavioral, and neuropsychological assessments. Heart rate monitoring, EFT and computer based cognitive tests (CANTAB) were supervised by the same investigator (SW) in all participants. In total, tests took ~2.5 h to complete. The girls were offered two breaks and soft drinks in between the testing blocks and a small financial compensation for participation in the study.

### Neuropsychological tests and questionnaires

#### Intellectual level

An abbreviated version of the *Wechsler Intelligence Scale for Children-III* (*WISC-III*) was used to generate an estimate of general cognitive ability. Two verbal (Vocabulary and Information) and two performance subtests (Block Design and Picture Completion) were used. This short-form combination has been shown to have a high reliability (Atkinson and Yoshida, 1989).

#### Memory tests

The *Rey Auditory Verbal Learning Test* [*RAVLT*; Dutch version: (Saan and Deelman, 1986)] was used to evaluate auditory-verbal memory. The recognition component was not assessed in this study. We derived five scores: Immediate Memory, Best Memory, Proactive Interference, Retroactive Interference, and Delayed Recall. We also computed two combined scores which are frequently used in studies that employ RAVLT: Learning Rate, reflecting the learning ability of the subject, and Total Learning, representing the capacity to recall and accumulate words across learning trials.

The *Continuous Visual Memory Test* (*CVMT*; Trahan and Larrabee, 1988) measures visual learning and memory, i.e., acquisition of information and retention over time (storage and retrieval). Acquisition or short-term memory included Immediate Memory and Proactive Interference scores, as well as Learning Rate score. Storage includes the CVMT Recognition score. Retrieval from long-term storage included Delayed Recall, Retroactive Interference, Best Learning, and Total Learning scores on both the RAVLT and CVMT.

#### Spatial ability

The *Mental Rotation Test* in which the subject was asked to compare two 3D objects and state if they are the same images (non-mirror or mirror images) was an adapted version of the task used by Hugdahl et al. (2006), originally developed by Shepard and Metzler (1971). The test had 20 pairs of images, the subjects were judged on how accurately, and rapidly they could distinguish between the pairs. The task has not been specifically validated for use in children, however in our sample the children performed similarly to what has been observed in adults.

#### Executive function and attention

A selection of four tests from the *Delis-Kaplan Executive Function System* (Delis et al., 2001) was used to assess different aspects of executive functions; the *Trail Making Test*, the *Verbal Fluency Test*, the *Color Word Interference Test*, and the *Design Fluency Test*. Composite executive functioning and processing speed domain scores are expressed as mean of subscale *z*-scores.

Additionally a selection of four tests from the Cambridge Neuropsychological Test Automated Battery (CANTAB), provided by Cambridge Cognition Ltd. was used to further assess executive function and attention. CANTAB tests are computerized, giving higher chance to discover minor differences between the groups. Although CANTAB tests were originally developed to assess patterns of cognitive decline in adults, their applicability for usage in children in age group 5–12 has been previously confirmed (Luciana and Nelson, 2002). The following tests were included:

*Choice reaction time (CRT)* is a 2-choice reaction time test with stimulus and response uncertainty introduced by having two possible stimuli (left and right arrows) and two possible responses (left and right buttons). Mean correct response latency and percentage of correct responses were recorded as outcome measures.
*Match to sample visual search (MTS)* is a matching test, with a speed/accuracy trade-off. The subject is presented with a sample-stimulus figure, composed of four colored elements displayed in the middle of the screen. After a brief delay, a varying number of similar patterns (1, 2, 4, or 8) are shown around the edge of the screen with only one of them matching the sample-stimulus pattern. The subject has to touch the matching pattern as fast as possible on the screen. Mean correct response time and percentage of correct responses were used as outcome measures.
*Spatial working memory (SWM)* tests subject's ability to retain spatial information and to manipulate remembered items in working memory. A number of colored boxes are shown on the screen. By process of elimination, the subject should find one blue “token” in each of a number of boxes. The number of boxes is gradually increased from three to eight boxes and the color and position of the boxes are changed from trial to trial to discourage the use of stereotyped search strategies. Total number of errors and SWM Search Strategy were used as outcome measures.
*Stop signal task (SST)* is a response inhibition test, giving a measure of an individual's ability to inhibit a pre-potent response. The subject is told to press the button that corresponds to the direction of the arrow presented on the computer screen, but, if they hear an auditory signal, they should withhold their response. There are five assessed blocks, each of 64 trials. The last four blocks were subjected to statistical analysis. The main outcome measure was the Stop Signal Response Time (SSRT), which is an estimate of the latency of the stop process. Additionally, the probability of inhibiting the response when signal occurred was calculated.

#### Parental questionnaires

The *Behavior Rating Inventory of Executive Function* (BRIEF)—the parent version (Dutch translation Smidts and Huizinga, 2009) assesses children's cognitive and behavioral aspects of executive function in home situations. It includes eight non-overlapping clinical scales (Inhibit, Initiate, Organization of Materials, Shift, Working Memory, Monitor, Emotional Control, Plan/Organize) and two validity scales (Negativity and Inconsistency of responses)

The *Child Behavior Check List*—4–18 years (Dutch translation: Verhulst and Van der Ende, 2004) is a standardized measure of academic, social competence, and behavioral problems. The questionnaire is completed by parents and includes eight sub-scales: Withdrawn, Somatic complaints, Anxious/Depressed, Social Problems, Thought Problems, Attention Problems, Delinquent Behavior, and Aggressive Behavior. The first three subscales add up to the Internalizing Problems scale and the last two to the Externalizing Problems scale. Finally, the overall Total Problems scale consists of all items. Additionally a Social Competence scale is derived from items grouped into Activities, Social, and School constructs. For each scale, *T*-scores (mean = 50 ± 10) can be obtained. A clinical cut-off point on the Total, the Internalizing and the Externalizing score was set at *T* = 60.

#### Socioeconomic indicators

Two socioeconomic indicators for parental occupations were used. An occupational class was constructed on the basis of International Standard Classification of Occupations (ISCO-08) (ILO, 1990) namely (1) managers and professionals; (2) technicians, clerks, and service workers; and (3) craft workers, machine operators, and elementary occupations. The number of years of formal education was divided into three groups: Secondary school, Higher education Short Type, and Higher Education Long Type or University.

### Emotion processing

#### The emotional flanker task (EFT)

**Figure 1:**
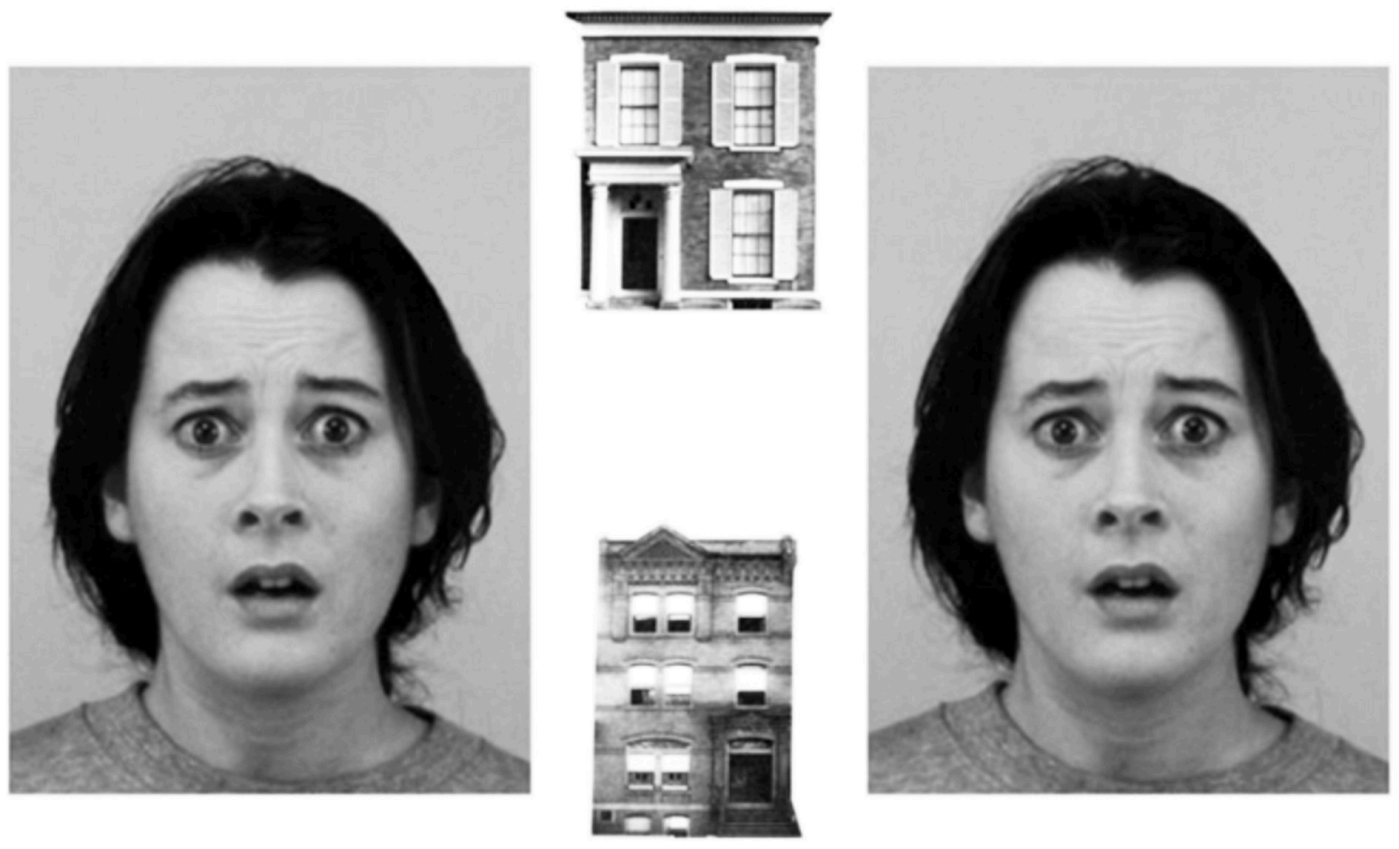
**Emotional flanker task**. In 207 trials, children were requested to decide as fast as possible whether two houses were identical or not. The faces were irrelevant for task solution and did not need to be attended to. The difference between reaction times in the presence of anxious and neutral faces (flanker valence effect) was used as a measure of emotional reactivity. Pictures of facial expressions were obtained from the Karolinska Directed Emotional Faces database (Lundqvist et al., 1998).

EFT was used to assess emotional reactivity. The task is an adapted version based on previous studies (Bishop et al., 2004). With an inter-trial interval of 1000 ms, on each trial, two faces and two houses were presented in horizontal and vertical pairs, respectively (Figure 1). Participants were instructed to decide as fast as possible whether the presented buildings were identical or not, and to respond by pressing a corresponding response button. They were informed that the faces presented in the periphery were irrelevant and didn't need to be attended to. If a participant did not make a choice within the first 4 s, the next trial was automatically presented. After five practice trials, participants were exposed to 207 trials, starting with three consecutive trials with neutral flankers to increase the effects of emotional flankers (Bishop et al., 2004). Out of the remaining 204 trials, target stimuli (houses) were identical in 50% of the trials; in 35% of all presentation trials flanker stimuli consisted of anxious faces, and in 65% of trials of faces with neutral expressions. The lower proportion of emotional flankers was chosen to increase the stimulus valence and resulting reactivity to these trials (Bishop et al., 2004).

The main outcome measure was a flanker-valence effect (FVE), which was calculated by subtraction of reaction times in the valence condition “neutral” from valence condition “anxious.” Larger reaction time differences between distractor valences “neutral” and “anxious” were interpreted as higher emotional reactivity (Grose-Fifer et al., 2013). To avoid confounding biases caused by different processing of identical and non-identical target stimuli, behavioral analyses were done separately for both conditions. Only correct responses were analyzed.

#### Heart rate and heart rate variability (HRV)

HRV has been extensively used in psychophysiological research to assess emotion regulation capacity (Thayer et al., 2012). Heart rate (HR) and HRV were calculated from the inter-beat intervals (IBIs), recorded with a sampling rate of 1000 Hz, using the *Polar RS800*® monitor. After a *Polar* belt was placed around the participant's chest, she was seated in a comfortable chair and asked to relax for 10 min (baseline). Thereafter, she was led over to a computer station and performed the EFT. The recording was stopped after task completion. Altogether, 20 min IBI-recordings of 29 out of 30 participants were collected. IBI recordings of one CPP girl were invalid due to equipment failure. Prior to analysis, all recordings were cleared of artifacts using ARTiiFACT software (Kaufmann et al., 2011). A minimum requirement of 95% of artifact-free IBIs was set as an inclusion criterion. No participant exceeded the 5% artifact-threshold; however there was a significantly higher mean number of artifacts in the treatment group (*M* = 7.1, *SD* = 4.4) vs. controls [*M* = 2.3, *SD* = 3.6; *t*_(27)_ = 3.2, *p* = 0.003]. Five minute-periods of data from the baseline and the EFT conditions, respectively, were chosen for further analyses, according to the Task Force (1996) guidelines (1996). HR and Root Mean Square of Successive IBIs (RMSSD) were analyzed as time domain measures. Additionally, power spectral density of High (HF), frequency was analyzed using Fast Fourier transformation following guidelines of the Task Force (1996). Frequency spectrum data were normalized by logarithmic transformation. Recordings from baseline and during EFT were analyzed separately.

### Statistical analysis

SPSS (version 20) was used for statistical analyses. Due to a relatively low number of participants, resampling of data, applying bias-corrected and accelerated bootstrapping technique (5000 resamples) was used to control for data stability. For comparisons between the groups, independent sample *t*-tests were applied, while a paired sample *t*-test was used to assess the differences between repeated measurements. If differences between groups were significant, Cohen's *d* was calculated for effect size estimation. Partial correlations were used to explore the associations between treatment duration and cardiac measures and behavioral test while controlling for chronological age. Group differences in socioeconomic status were assessed by comparing the educational levels of children's parents using Fisher's exact test.

## Results

The clinical characteristics of CPP girls and controls are summarized in Table 1. Eleven out of 15 girls had started treatment with 11.25 mg intramuscular injection of GnRHa (Decapeptyl SR®) every 10th week, and 4 out of 15 girls with a 3.75 mg injection every 4th week. Patients were monitored regularly and their medication was adjusted in case of incomplete pubertal suppression as judged by physical examination and LH/FSH blood sampling or repeated GnRH testing; 11.25 mg every 8 weeks in three girls, 11.25 mg every 6 weeks in one girl, 11.25 mg every 10 weeks in two girls (from 3.75/per 4 weeks), and 11.25 mg every 12 weeks in one girl. At study entry (T1), 14 out of 15 girls had a Tanner score for breast development equal to or less than at the start of the treatment. Median duration of GnRHa treatment was 28 months (range: 8–57) at the time of the study. As expected, body height, BMI and bone age were still higher in treated CPP girls as compared to controls. Over the course of treatment, the difference between bone age (BA) and chronological age (CA) was reduced by 8.6 months, [*t*_(14)_ = 2.2, *p* = 0.042]. Whereas all control girls were healthy, one CPP girl suffered from chronic otitis media and one from hip dysplasia, independently of the CPP and GnRHa treatment.

**Table 1:** **Clinical characteristics of 15 girls with central precocious puberty (CPP) at the onset of gonadotropin releasing hormone analog therapy (T0) and at the moment of the study (T1), compared to 15 age-matched controls**.

**Clinical characteristics**	**CPP (T0)**	**CPP (T1)**	**Control**	***p***
CA (years)	7.5 (4.4; 9.8)	10.4 (9.2; 11.8)	10.3 (9.1; 11.4)	0.877
Height (*z*-score)	0.9 (−0.7; 3,1)	0.8 (−0.4; 3.2)	0.3 (−2.8; 1.6)	0.023
Weight (*z*-score)	0.7 (−0.8; 2.4)	0.8 (0.0; 2.5)	−0.5 (−2.0; 1.2)	0.001
BMI (kg/m^2^)	17.0 (15.0; 30.0)	18.0 (15.0; 34.0)	16.0 (13.0; 20.0)	0.055
BA (years)	9.4 (7.8; 13.0)	11.5 (10.0; 13.0)	9.3 (7.8; 12.0)	< 0.001
Δ BA−CA	2.0 (0.3; 4.8)	1.3 (−0.4; 3.2)	−1.0 (−2.0; 1.7)	< 0.001
Tanner stage M (n) and P (n)		M1 = 6; P1 = 4	M1 = 10; P1 = 14	
		M2 = 3; P2 = 5	M2 = 3; P2 = 0	
		M3 = 6; P3 = 5	M3 = 2; P3 = 1	
		M4 = 0; P4 = 1	M4 = 0; P4 = 0	
**Socio-economic background**		**CPP (T1)**	**Control**	
**EDUCATION**				
Mother: S/H1/H2		47/33/20%	23/62/15%	0.307
Father: S/H1/H2		33/47/20%	29/57/14%	0.842
**OCCUPATION MOTHER**				
Manager or professional		27%	69%	
Technicians, clerks and service workers		60%	31%	
Craft workers, machine operators, elementary occupations		13%	0%	
**OCCUPATION FATHER**				
Manager or professional		33%	38%	
Technicians, clerks and service workers		40%	31%	
Craft workers, machine operators, elementary occupations		27%	31%	

*The values are presented as medians (min; max); CPP (T0), CPP group at the time of diagnosis; CPP (T1), CPP group at the time of study entry; CA, chronological age; BMI, body mass index; BA, bone age; Δ BA–CA, difference between bone age and chronological age in years; Tanner stage M(n) and P(n), Tanner stage for breast (M) and pubic hair (P) development; S/H1/H2, percentage of parents who fulfilled secondary school (S)/higher education short type (H1)/higher education long type (H2); p, significance level (independent sample t-test) of difference between CPP (T1) and Control for continuous variables and Fisher's exact test for education*.

### Neuropsychological findings

Table 2 summarizes the results of the neuropsychological assessment. The mean estimated IQ was 94 (range: 73–116) for CPP girls and 102 (range 81–125) for control girls; the difference was not significant. The estimated IQ scores were consistent with the school situation; 26/30 girls were attending an appropriate grade for their age. Two girls from the control group and two girls from the CPP group were delayed by 1 year at school. No associations were found between IQ scores and duration of GnRHa therapy. The statistical comparison of parental educational level (Fisher's exact test) showed no significant difference between groups (Table 1).

**Table 2:** **Cognitive function of girls with central precocious puberty (CPP) treated with gonadotropin releasing hormone analog therapy, compared to age-matched controls**.

	**CPP *N* = 15**	**Controls *N* = 15**	***p***
**IQ (WISC III)**			
Total IQ	94.1 (12.1)	101.9 (12.0)	0.09
Performance	96.8 (7.8)	103.2 (11.2)	0.08
Verbal IQ	98.4 (10.7)	101.6 (9.4)	0.39
WISC information	10.9 (2.2)	10.7 (1.9)	0.80
WISC vocabulary	9.1 (2.6)	10.5 (1.7)	0.09
WISC incomplete pictures	7.7 (3.2)	10.1 (4.0)	0.07
WISC bloc design	8.5 (3.0)	9.8 (3.4)	0.30
**MEMORY**			
**Verbal (RAVLT)**			
Trial 1 (immediate memory)	6.2 (1.9)	7.1 (1.9)	0.23
Trial 2	9.0 (2.4)	10.2 (2.7)	0.22
Trial 3	9.8 (3.7)	11.7 (1.6)	0.08
Trial 4	12.3 (1.9)	13.2 (2.1)	0.21
Trial 5 (best memory)	12.7 (1.9)	12.9 (2.2)	0.80
Learning rate	6.5 (1.8)	5.9 (2.2)	0.38
Total learning	50.1 (9.3)	55.1 (8.5)	0.13
Proactive interference	5.7 (1.1)	6.1 (2.9)	0.70
Retroactive interference	11.7 (2.2)	11.3 (2.9)	0.62
Delayed recall	11.1 (3.4)	11.1 (2.7)	1.00
**Visual (CVMT)**			
Total score	108.7 (18.3)	114.4 (16.1)	0.40
Recognition	4.2 (1.8)	4.7 (1.2)	0.42
**MENTAL ROTATION**			
Number correct (out of 20)	14.6 (2.4)	14.3 (2.3)	0.77
Total time (s)	160.3 (63.3)	189.3 (62.3)	0.22
**COGNITIVE EXECUTIVE FUNCTION**			
Trail Making Test: shifting	9.3 (3.7)	11.4 (2.5)	0.07
Color-Word Interference Test: shifting	11.2 (2.4)	10.5 (3.0)	0.50
Color-Word Interference Test: interference	11.3 (2.2)	11.5 (3.5)	0.86
Verbal Fluency Test: shifting	12.9 (2.9)	12.6 (3.3)	0.77
Design Fluency Test: shifting	12.1 (2.1)	12.3 (3.2)	0.79
Composite *z*-score	−0.09 (0.9)	0.09 (1.1)	0.64
**PROCESSING SPEED**			
Trail Making Test: number sequencing	8.0 (3.3)	10.9 (2.2)	**0.01**
Trail Making Test: letter sequencing	9.4 (3.1)	9.4 (3.1)	1.00
Color-Word Interference Test: color reading	10.0 (2.6)	10.5 (2.7)	0.59
Color-Word Interference Test: word reading	11.2 (1.4)	11.3 (2.3)	0.85
Verbal Fluency Test: category fluency	11.7 (2.9)	12.1 (2.8)	0.72
Verbal Fluency Test: letter fluency	8.9 (1.9)	8.3 (2.1)	0.42
Design Fluency Test: filled dots	10.4 (3.0)	11.7 (2.0)	0.19
Design Fluency Test: empty dots	11.3 (2.7)	12.3 (3.9)	0.45
Composite *z*-score	−0.20 (1.1)	0.2 (0.9)	0.29
**ATTENTION AND EXECUTIVE FUNCTION (CANTAB)**			
Choice reaction time: correct responses (%)	98.4 (1.3)	99.0 (1.1)	0.18
Choice reaction time: mean latency (ms)	469.6 (107.9)	492.4 (112.1)	0.57
Match to sample visual search: correct responses (%)	98.1 (2.0)	98.9 (1.7)	0.23
Match to sample visual search: mean latency (s)	3.4 (0.7)	3.7 (1.0)	0.29
Spatial working memory: number of errors	30.7 (14.6)	26.5 (19.3)	0.50
Spatial working memory: search strategy	32.7 (5.4)	34.1 (6.2)	0.52
Stop signal task: successful stops (%)	47.3 (7.4)	50.1 (9.8)	0.30
Stop signal response time (ms)	205.8 (53.2)	214.9 (55.1)	0.64

*IQ estimations are presented as standardized IQ scores (normative mean = 100, SD = 15). WISC subscales, Cognitive executive function and Processing speed are presented as scaled scores (normative mean = 10, SD = 3). CANTAB, Memory, and Mental rotation tests results are presented as raw scores; WISC, Wechsler Intelligence Scale for Children; RAVLT, Rey Auditory Verbal Learning Test; CVMT, Continuous Visual Memory Test; CANTAB, Cambridge Neuropsychological Test Automated Battery; p, significance level (independent sample t-test) of difference between CPP and controls. p < 0.05 are marked in bold*.

Regarding verbal (RAVLT) and non-verbal memory tests (CVMT), both groups performed very similarly, showing no significant differences. The four CANTAB tests targeting attention and executive function are sensitive to small differences in performance. Nevertheless, both groups showed very similar scores on all four tests, showing no significant differences. There were no significant between-group differences on the composite *z*-scores of cognitive executive function and processing speed except for the Trail Making Test-Number Sequencing, where CPP girls performed worse than controls [*t*_(28)_ = 2.8, *p* = 0.01, *d* = 1.32]. The BRIEF questionnaire scores showed no significant differences regarding parental reported executive function (Table 3).

**Table 3:** **Cognitive executive function and behavioral problems in girls with central precocious puberty (CPP) treated with gonadotropin releasing hormone analog therapy, compared to age-matched controls**.

	**CPP *N* = 14**	**Control *N* = 10**	***p***
**BRIEF**			
**Clinical scales**			
*Behavioral regulation index*	51.8 (11.7)	47.6 (7.7)	0.33
Inhibit	52.1 (9.8)	48.9 (10.6)	0.47
Shift	52.7 (10.7)	46.7 (6.9)	0.14
Emotional control	50.1 (10.6)	48.3 (6.8)	0.65
*Metacognition index*	50.7 (10.1)	49.1 (10.4)	0.71
Initiate	50.5 (7.2)	46.5 (5.3)	0.15
Working memory	52.5 (11.0)	47.7 (9.0)	0.26
Plan/organize	49.1 (9.0)	49.7 (9.1)	0.88
Organization of materials	48.3 (7.7)	49.5 (11.2)	0.77
Monitor	48.7 (7.1)	50.3 (8.8)	0.62
*Global executive composite*	51.3 (9.8)	48.1 (10.5)	0.46
**Validity scales**			
Negativity	0.5 (0.9)	0.2 (0.6)	0.36
Inconsistency of Responses	2.0 (1.4)	1.9 (1.7)	0.90
	***N*** = **15**	***N*** = **13**	
**CBCL**			
*Total social competence*	44.5 (11.6)	47.0 (6.4)	0.49
Activities	41.4 (9.0)	44.5 (6.6)	0.31
Social	45.9 (7.4)	47.7 (6.3)	0.51
School	48.8 (9.1)	51.3 (6.1)	0.41
*Internalizing problems*	56.7 (10.9)	53.5 (11.1)	0.45
Withdrawn	55.2 (8.3)	55.2 (6.6)	0.99
Somatic complaints	58.8 (9.0)	54.8 (6.2)	0.18
Anxious/Depressed	58.1 (9.8)	56.3 (8.5)	0.61
*Externalizing problems*	48.6 (10.3)	45.7 (7.7)	0.41
Delinquent behavior	53.5 (5.7)	52.9 (5.6)	0.78
Aggressive behavior	53.0 (6.0)	51.2 (2.6)	0.33
*Total problems*	52.3 (11.5)	48.3 (10.2)	0.34
Social problems	54.1 (8.5)	52.7 (5.6)	0.62
Thought problems	53.7 (6.7)	52.7 (6.7)	0.68
Attention problems	55.7 (6.7)	55.7 (10.6)	0.99

*Except for validity scales, all values are presented as T-scores (normative mean = 50, SD = 10). BRIEF, Behavior Rating Inventory of Executive Function questionnaire, parent version; CBCL, Child Behavior Check List questionnaire, parent version; p, significance level (independent sample t-test) of difference between CPP and controls*.

### Behavioral and emotional problems (CBCL)

Overall, the CBCL results (Table 3) showed that CPP girls did not have significantly more behavioral problems than controls and they displayed similar social competence. When compared to normal range (*T* = 50 ± 10), the most elevated scores were observed within *internalizing problems* domain on *withdrawn, somatic complaints* and *anxious/depressed* subscales. Out of 15 CPP girls, two had elevated scores at a clinically meaningful level (*T* > 60) on all of these four scales apart from somatic complains where four out of 15 had a *T-*score > 60. Similarly, in the control group two girls showed elevated *T*-scores on each of the same scales.

### Emotional reactivity

Mean reaction times in EFT for all four (2 × 2) conditions (range: 1062–1319 ms) were comparable to adult data of similar versions of this task (Bishop et al., 2004). In trials with non-identical targets (mismatch-condition) reaction times were generally slower, although not significantly, in both groups compared to identical target condition (data not shown). Interestingly, for the non-identical target condition the main outcome measure, calculated as difference in reaction times in presence of “neutral” and “anxious” faces (FVE), showed a significant distraction-related slow-down of motor response in the CPP group (FVE = 36.9 ms, *SD* = 93.3), whereas response facilitation was seen in controls [FVE = −42.7 ms, *SD* = 75.5; *t*_(28)_ = −2.6, *p* = 0.016, *d* = 1.04]. In trials with identical targets, the groups did not differ significantly in their distractibility, showing distraction related response facilitation in both the control (FVE = −8.3 ms, *SD* = 68.6) and the CPP group [FVE = −71.7, *SD* = 83.4; *t*_(28)_ = 1.2, *p* = 0.238].

### Heart rate and heart rate variability

Under resting conditions, HR was significantly lower in the CPP group (HR = 76.4/min, *SD* = 5.5) as compared to controls [HR = 87.7/min, *SD* = 10.9; *t*_(27)_ = 3.5, *p* = 0.004, *d* = −1.03]. HRV parameters showed significantly higher values for the CPP group as compared to controls: RMSSD 72.4 (*SD* = 19.1) vs. 43.8 (*SD* = 19.7), *t*_(27)_ = −3.9, *p* = 0.004, *d* = 1.44, and Ln(HF); 7.5 (*SD* = 0.6) vs. 6.6 (SD = 1.0), *t*_(27)_ = −3.1, *p* = 0.004, *d* = 0.95. The same pattern was evident when participants performed the EFT paradigm (data not shown).

#### Effect of treatment duration

**Figure 2:**
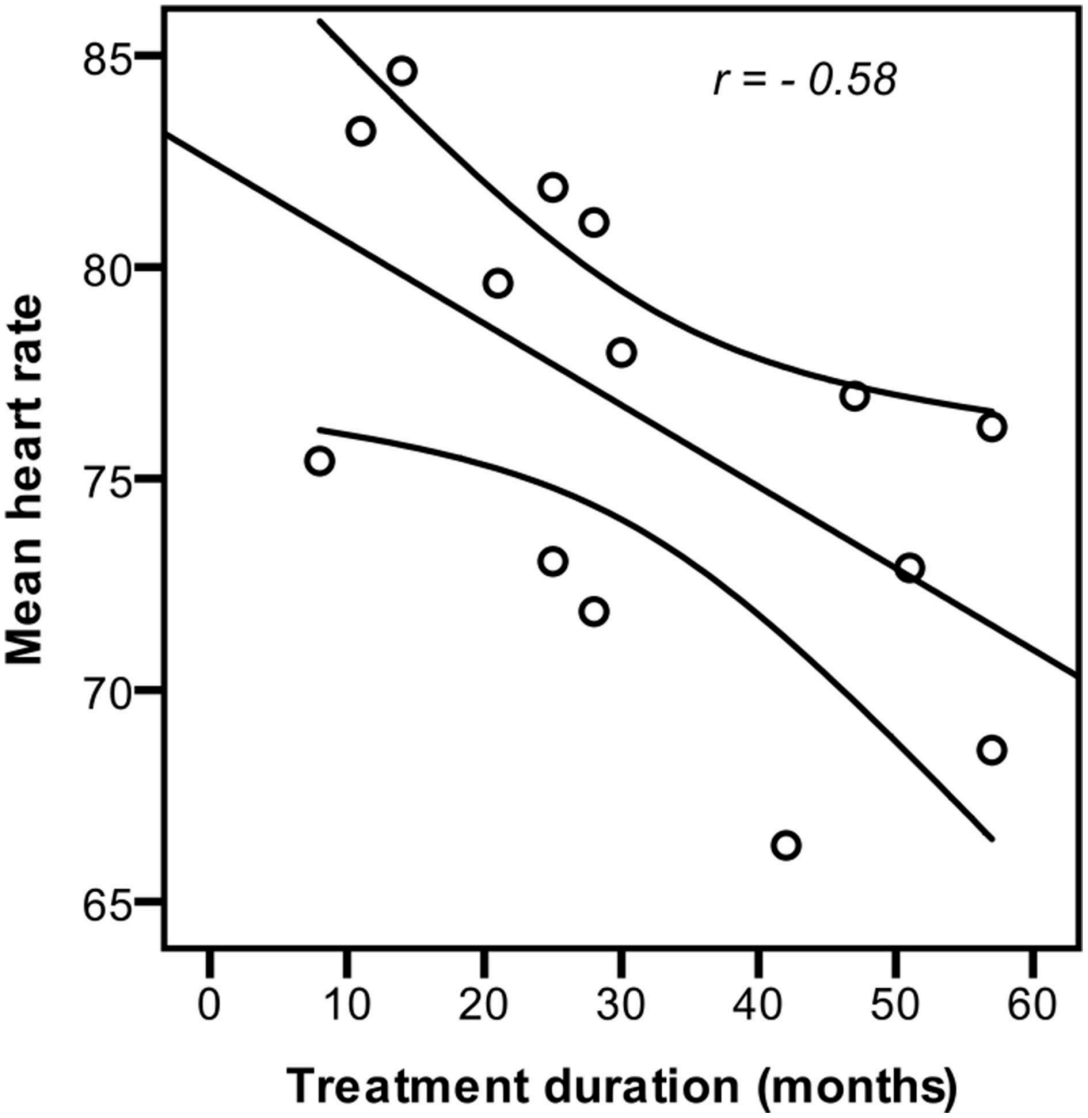
**Influence of treatment duration on heart rate**. Longer GnRHa treatment duration was significantly correlated (*p* = 0.037) with lower resting heart rate (controlled for age) in girls with idiopatic central precocious puberty.

Partial correlation (controlled for age) between treatment duration and heart rate revealed that longer treatment duration was associated with lower mean heart rate, *r* = −0.58, *p* = 0.037 (Figure 2). No significant correlations between treatment duration and any of the HRV or EFT measures were found.

### *Post-hoc* power analyses

In order to estimate the probability of falsely rejecting the null hypothesis, the power of the study was calculated *post-hoc*. The majority of the neuropsychological tests and questionnaires used in this study refer to norm data expressed in form of standardized, scaled or T scores, where a difference of more than one standard deviation from the population mean is considered to be clinically significant. Consequently, for independent sample *t*-test, statistical power (1–β) was found to be 0.75, based on an α level of 0.05 and a difference of one standard deviation between the groups (Faul et al., 2007).

## Discussion

The main objective of the study was to assess psychological functioning in GnRHa treated girls with idiopathic CPP as compared to age-matched controls. With respect to cognitive functioning, behavioral, and social problems, treated CPP girls do not differ from age matched controls. However, the significance of the results regarding emotional reactivity and emotional regulation capacity remains unclear. The interpretation of HRV findings, as a measure of emotional regulation capacity, is complicated by the fact that GnRHa may directly influence heart rhythm through GnRH cardiac receptors (Dong et al., 2011). Possible interpretations of our findings as well as methodological challenges are discussed below.

### Cognitive functioning and psychosocial problems

In contrast to previous reports on elevated verbal *IQ scores*, and/or accelerated school achievements in CPP girls (Galatzer et al., 1984; Ehrhardt and Meyer-Bahlburg, 1986), the GnRHa treated CPP girls' estimated IQ in the current study was within the normal range and somewhat lower, although not significantly, than that of controls (Table 1).

No significant differences between the CPP and the control group were seen with regard to *cognitive performance* neither on paper and pencil nor in computer based tests concerning memory, spatial ability, attention, and executive functions. Only in the Trail Making Test—Number Sequencing, assessing processing speed, the CPP group showed significantly poorer performance (Table 2). This finding is difficult to explain since neither the very similar Trail Making Test—Letter Sequencing, nor any other of the processing speed tests showed significant differences between the groups. Taking into account that the *p*-values were not corrected for multiple testing, it is possible that this finding is accidental. In line with this, the CPP girls' parents did not report any problems with regard to executive functioning as measured by the BRIEF questionnaire.

*Behavioral problems and social competence* were assessed with the parent version of the CBCL and showed no statistical differences between the groups (Table 3). Several earlier studies reported different levels of problems in CPP using the CBCL. Based on CBCL scores of 33 girls with CPP compared to age matched controls, Sonis et al. (1985) concluded that overall they could be described as troubled, depressed, aggressive, socially withdrawn, and moody (Sonis et al., 1985). In their results, the authors did not discriminate between the idiopathic and other types of CPP. Moreover, at the time of CBCL scoring, only 8 out of 33 girls received GnRHa treatment, which at the beginning of the 1980s was still considered to be experimental; the results did not discriminate between the treated and untreated girls. In a longitudinal study, Xhrouet-Heinrichs et al. (1997) followed 20 girls with CPP (15 of idiopathic type) of which 15 were treated with GnRHa for 2 years. The CBCL was filled out by parents at 1 and 24 months after diagnosis. Authors reported elevated *withdrawal, anxious/depressed*, or *aggressive behavior* scores in 30–40% of the girls. After 2 years, most of the same girls still displayed elevated scores. In contrast, in our study, only 2 out of 15 (13%) CPP girls showed elevated scores on the same scales, which was very similar to what we observed in age matched controls (2 out of 13). In a more recent study, Kim and Lee (2012) compared 34 girls with idiopathic CPP (mean age 8.1) to 39 same age controls; no information about whether CPP girls were treated was provided. The results showed that although CPP girls had more behavioral problems, no significant differences were observed between the two study groups in terms of clinically important scores.

While all of the presented studies differ with respect to the age of participants, treatment duration, and assessment time, it is worth mentioning that the most recent studies show less psychosocial problems in CPP children than older ones. Although speculative, the decrease in psychosocial problems reported in recent studies (including this one) could be a result of less stigmatization of and more openness about this condition and perhaps the modified management of CPP with regard to treatment initiation and monitoring.

### Emotional processing

#### Emotional reactivity

In the presence of fearful-faces in the mismatch-condition, CPP girls showed increased reaction times compared to neutral-faces, while the opposite was seen in the control group; in statistical terms this difference represented a large effect (Cohen, 1992). However, no significant differences between the groups were observed in the matched-condition.

In healthy samples, emotional stimuli can facilitate interference resolution, and enhance task performance by reducing reaction times (Levens and Phelps, 2008). In contrast, in vulnerable groups, increased reactivity toward emotionally intensive stimuli can be associated with increased interference between emotions and executive functions, leading to increased reaction times in a seemingly unrelated choice reaction task (for an overview see Mueller et al., 2011). Increased reaction time in the CPP group may therefore indicate higher distractibility by task-irrelevant stimuli (anxious faces) and increased interference with executive functions because processing of emotional (particularly negative) stimuli may impair executive motor control (de Houwer and Tibboel, 2010; Herbert and Sütterlin, 2011). Nevertheless, the fact that the same pattern was not observed in a matched-condition somewhat weakens such interpretation. While it is true that the mismatch-condition represents a higher cognitive demand, expressed by a tendency toward slower reaction times in both groups when compared to the matched-condition, we cannot firmly conclude that this minor difference in cognitive load is responsible for the diversity of outcomes. In summary, although part of the findings suggest differences in emotional reactivity between the groups, the results are not conclusive.

#### Cardiac function and emotional regulation

GnRHa treated CPP girls had significantly lower resting HR and significantly higher HRV than controls. Resting HR was negatively correlated with treatment duration, i.e., longer GnRHa treatment was associated with lower resting HR (Figure 2), while no correlations between HRV and treatment duration were found. The results indicate large effects; Cohen's *d* > 0.8 and Pearson's *r* > 0.5 (Cohen, 1992).

The main goal of the heart rhythm recording was estimation of HRV as a proxy for cardiac vagal influence (Thayer et al., 2012). Consequently, the lower HR and higher HRV could suggest that treated CPP girls have better emotion regulation capacity and higher adaptability to changing contexts than controls. However, for such interpretation to be valid, a direct GnRHa effect on heart rhythm should be excluded. Such effect could be possibly mediated via GnRH receptors that have been found in cardiomyocytes (Kakar and Jennes, 1995). It has been shown in a murine model that GnRH can augment cardiomyocytes' contractile characteristics via a GnRH receptor/phosphokinase A-dependent (PKA) mechanism, while the opposite effect was observed after administration of GnRH receptor blocker (Dong et al., 2011). Although there is no direct evidence of such effects in humans, findings from other studies might be attributed to these cellular mechanisms; prolonged electrocardiographic QT intervals were recorded in GnRHa treated prostate cancer patients (Garnick et al., 2004), and GnRHa therapy in young women with symptomatic uterine leiomyoma, endometriosis, or candidates for *in vitro* fertilization led to significantly reduced peak flow velocity and cardiac index (stroke volume × heart rate; Eckstein et al., 1993).

A possibility of direct GnRHa effect on heart rhythm makes interpretation of the HRV results difficult, since HRV is only a proxy for central, prefrontally mediated inhibitory processes that are peripherally expressed through cardiac vagal influence (Thayer et al., 2012). At the planning stage of this study, the possibility of such interactions had not been described. If further confirmed, these findings can make applicability of HRV as a measure of emotional regulation capacity invalid in individuals receiving GnRHa treatment.

#### Assessment of emotional processing—conclusions and further steps

Overall, our findings do not provide firm conclusions with regard to differences in emotional processing between the GnRHa treated CPP girls and age-matched controls. The diversity of the results suggests that more emphasis should be put on the investigation of emotion processing in future studies. In this respect both psychophysiological and experimental paradigms that tap in to the different domains of emotional processing and regulation (i.e., capacity, reactivity, recovery, and sensitivity) should be considered.

### Methodological considerations

Psychological functioning of GnRHa treated CPP girls may depend on a number of different mechanisms including direct effects of GnRHa on the brain, cessation of sex steroid influences, degree of exposure to the pubertal hormones before treatment initiation, the course of the CPP condition itself or psychosocial/educational environment. It is thus difficult to isolate the impact of GnRHa treatment on psychological functioning. The most appropriate study design to discriminate between GnRHa effects and other factors would be a randomized controlled trial (RCT). Since a RCT cannot be conducted due to ethical reasons, the most obvious alternatives include comparison of treated CPP children and controls matched for either chronological or biological age, in a cross-sectional or a longitudinal study. Comparison of cognitive development trajectories of non-CPP control and CPP-treatment groups through several measurement points, i.e., pre-, under- and post-treatment can provide most hints about GnRHa treatment impact on cognitive development. Nevertheless, while providing more information, the longitudinal design still cannot ensure proper isolation of GnRHa influence on brain development from the natural course of the condition, including pretreatment sex steroid exposure. Furthermore, the question remains if matching should be done by chronological or biological age. It can be argued that matching by chronological age is not appropriate since CPP children's biological age is higher than that of their chronological age peers. Matching by biological age would ensure comparable levels of biological maturation between the groups, which theoretically could increase the likelihood that the observed cognitive differences are indeed related to the actions of GnRHa. On the other hand, development of cognitive functioning cannot be separated from environmental influences. The majority of the GnRHa treated CPP girls attend school classes that are appropriate to their chronological age and socialize with the same age peers. It is therefore, in our opinion, more ecologically valid to evaluate cognitive functioning in comparison to the same chronological age population.

Finally, to gain mechanistic insights into the GnRHa effects on brain development, animal studies might provide further knowledge. Our group has previously conducted a twin sheep RCT where one of the twins had their puberty blocked with GnRHa. The results indicated that GnRHa might have influenced the development of cognitive functions related to emotion processing, while no clear effects on cognitive functions that did not involve emotional processing were found (Wojniusz et al., 2011, 2013; Evans et al., 2012; Nuruddin et al., 2013a,b,c; Robinson et al., 2014). While this study represented a delayed rather than precocious puberty model and translation of the results to humans should be made with caution, it suggests emotional processing as a potential area of GnRHa influence on the brain.

### Sample size and limitations

Due to the low number of CPP patients receiving GnRHa treatment, only 15 CPP girls were included in this study, which can limit its statistical power. Nevertheless, *post-hoc* power analysis showed 1 − β to be 0.75, which gives a fair chance of rejecting the false null hypothesis taking into account a group difference of interest of 1 *SD*. We argue that with regard to most of the cognitive tests and questionnaires used in this study, particularly those with known norm data, 1 *SD* represents a boundary of what is a clinically interesting difference. Although more participants would increase the statistical power and possibility of discovering smaller group differences, in our opinion, the present study provides useful information and suggestions for future research areas in a field that to date has been rarely investigated.

Regarding experimental and physiological measures for assessment of emotion processing, in the hindsight, we did not fully succeed in our choice of methods. While the results of EFT were ambiguous, perhaps depending on motivational factors, and overall difficult to interpret, the HRV findings were possibly not even valid as a proxy of cardiac vagal influence (see Section Cardiac Function and Emotional Regulation). Alternative approaches that could be applied in future studies could include functional neuro-imaging techniques to detect subtle changes of emotion processing directly within the central nervous system rather than applying peripheral proxies. Alternative behavioral measures of emotion-related attentional processing could be obtained via more implicit approaches that are less confounded by motivational states (e.g., eye-tracking).

### Conclusion

Overall, the findings suggest that GnRHa treated CPP girls do not differ in their cognitive functioning, behavioral, and social problems from the same age peers, at least, in settings that do not involve emotional processing. Although our findings with regard to emotional regulation and reactivity are inconclusive, they provide hints that CPP girls may differ in these areas from same age peers. We, therefore, suggest that future studies should to a higher degree emphasize investigation of emotional processing in a CPP population.

Finally, the differences in cardiac rhythm, expressed as lower HR in the CPP group and the fact that they were increasing with treatment duration, should be more closely followed up in the future, making use of methodologies that are appropriate for investigation of cardiac health.

## Author contributions

SW, One of the designers of the study; collected the data with regard to CANTAB, HRV, and EFT tests; analyzed the data from these experiments and drafted the manuscript with regard to introductory part, methods and analysis of CANTAB, HRV, and EFT, and drafted the discussion. NC, One of the designers of the study; main responsibility for subject recruitment; collected and analyzed the data with regard to neuropsychological tests and behavioral questionnaires; drafted the parts of the article associated with neuropsychological tests and behavioral questionnaires. SS, Took part in the data analysis and interpretation of results with regard to HRV and EFT experiments; critically reviewed the data analysis and the manuscript. SA, Responsible for the design of neuropsychological test battery; critically reviewed the data analysis and the manuscript. JD, IG, JV, KD, SV, MCr, Participated in subject recruitment/treatment; critically reviewed the data analysis and the manuscript. CV, Participated in design of the study; critically reviewed the data analysis and the manuscript. MCo, One of the designers of the study; participated in subject assessment/recruitment/treatment; critically reviewed the data analysis and the manuscript. IH, Primary investigator; responsible for the design of the study; critically reviewed the data analysis, and the manuscript.

## Funding

The study has been funded by the Department of Medical Neurobiology, Oslo University Hospital, Norway.

### Conflict of interest statement

The authors declare that the research was conducted in the absence of any commercial or financial relationships that could be construed as a potential conflict of interest. The reviewer, MK, and handling Editor declared their shared affiliation, and the handling Editor states that the process nevertheless met the standards of a fair and objective review.
